# Improved Transient Response Estimations in Predicting 40 Hz Auditory Steady-State Response Using Deconvolution Methods

**DOI:** 10.3389/fnins.2017.00697

**Published:** 2017-12-12

**Authors:** Xiaodan Tan, Qiuyang Fu, Han Yuan, Lei Ding, Tao Wang

**Affiliations:** ^1^School of Biomedical Engineering, Southern Medical University, Guangzhou, China; ^2^Department of Otolaryngology, Guangdong Second Provincial General Hospital, Guangzhou, China; ^3^Stephenson School of Biomedical Engineering, University of Oklahoma, Norman, OK, United States; ^4^College of Big Data and Internet, Shenzhen Technology University, Shenzhen, China

**Keywords:** auditory steady-state response, linear superposition hypothesis, multi-rate steady-state average deconvolution, continuous loop averaging deconvolution, stimulus sequencing scheme

## Abstract

The auditory steady-state response (ASSR) is one of the main approaches in clinic for health screening and frequency-specific hearing assessment. However, its generation mechanism is still of much controversy. In the present study, the linear superposition hypothesis for the generation of ASSRs was investigated by comparing the relationships between the classical 40 Hz ASSR and three synthetic ASSRs obtained from three different templates for transient auditory evoked potential (AEP). These three AEPs are the traditional AEP at 5 Hz and two 40 Hz AEPs derived from two deconvolution algorithms using stimulus sequences, i.e., continuous loop averaging deconvolution (CLAD) and multi-rate steady-state average deconvolution (MSAD). CLAD requires irregular inter-stimulus intervals (ISIs) in the sequence while MSAD uses the same ISIs but evenly-spaced stimulus sequences which mimics the classical 40 Hz ASSR. It has been reported that these reconstructed templates show similar patterns but significant difference in morphology and distinct frequency characteristics in synthetic ASSRs. The prediction accuracies of ASSR using these templates show significant differences (*p* < 0.05) in 45.95, 36.28, and 10.84% of total time points within four cycles of ASSR for the traditional, CLAD, and MSAD templates, respectively, as compared with the classical 40 Hz ASSR, and the ASSR synthesized from the MSAD transient AEP suggests the best similarity. And such a similarity is also demonstrated at individuals only in MSAD showing no statistically significant difference (Hotelling's *T*^2^ test, *T*^2^ = 6.96, *F* = 0.80, *p* = 0.592) as compared with the classical 40 Hz ASSR. The present results indicate that both stimulation rate and sequencing factor (ISI variation) affect transient AEP reconstructions from steady-state stimulation protocols. Furthermore, both auditory brainstem response (ABR) and middle latency response (MLR) are observed in contributing to the composition of ASSR but with variable weights in three templates. The significantly improved prediction accuracy of ASSR achieved by MSAD strongly supports the linear superposition mechanism of ASSR if an accurate template of transient AEPs can be reconstructed. The capacity in obtaining both ASSR and its underlying transient components accurately and simultaneously has the potential to contribute significantly to diagnosis of patients with neuropsychiatric disorders.

## Introduction

The auditory steady-state response (ASSR) elicited by periodical auditory stimulation is a major approach in clinic for hearing screening and frequency-specific hearing assessment (e.g., Silva et al., 2013; Francois et al., 2016). Recently, increasing number of studies have found abnormalities of ASSR in psychiatric disorders such as schizophrenia (O'Donnell et al., 2013; Thune et al., 2016), autism (Gandal et al., 2010), and bipolar disorder (Rass et al., 2010; Oda et al., 2012; Isomura et al., 2016). These findings suggest that ASSR could be a valuable biomarker in diagnosing various neurological and psychiatric disorders (McFadden et al., 2014; Thune et al., 2016). Despite these findings, the application of ASSR is still experiencing major problems due to the lack of knowledge and intensive debates regarding the underlying mechanism of ASSR (Ross et al., 2005; Bohorquez and Ozdamar, 2008; Presacco et al., 2010; Lutkenhoner and Patterson, 2015; Tan et al., 2015; Lutkenhoner, 2016).

ASSRs, recorded from the human scalp, have been found remarkably pronounced at 40 Hz as compared with other stimulus rates (Picton et al., 2003). Such an enhancement can be explained by the in-phase superimposition of a sequence of transient auditory evoked potentials (AEPs) elicited by individual stimuli. In fact, the major middle latency response (MLR) components follow tightly, such as Pa occurring at ~25 ms, behind the early response components usually termed as auditory brainstem responses (ABRs) (Galambos et al., 1981; Stapells et al., 1984). Proximity of these components and matching time intervals (25 ms equal to time interval among individual stimuli at 40 Hz stimulus rate) strongly support the linear superposition theory accounting for the generation mechanism of ASSR. The theory describes that ASSR is the linear mixing of multiple underlying transient AEPs each evoked by a stimulus (e.g., a click) periodically presented in a sequence. Therefore, based on the theory, if assuming each transient AEP is same, it can then be used as a template for the transient response of each stimulus to reconstruct ASSR, together with the timing of individual stimuli in the stimulus sequence.

Based on the superposition theory, one may expect that the ASSR synthesized from transient AEP templates would accurately predict the actual classical ASSR from recordings. However, the sporadic reports on the predictive discrepancy suggest other mechanisms may account for the ASSR generations. For example, Suzuki et al. (1994) reported that the prediction failed for subjects in the sleep state while it succeeded in waking state. Azzena et al. (1995) reported a phenomenon of over-prediction in peak-to-peak amplitudes occurring at rates higher than 40 Hz and under-prediction at rates < 40 Hz. In addition, an animal model also found insufficient accuracy for synthesized ASSRs using data directly from rat temporal cortex (Conti et al., 1999).

Some investigators thus proposed alternative mechanisms for ASSR, such as the theory of entrainment of a neuronal rhythm, i.e., phase synchronization (Ross et al., 2005; Thut et al., 2011; Lutkenhoner and Patterson, 2015). For example, Ross et al. (2005) challenged the superposition theory using a special designed stimulation protocol, which contained a 40 Hz amplitude-modulated regular sound and a separate channel of brief burst serving as a perturbing stimulus presented monaurally and dichotically. They found that regular ASSR attenuation caused by the burst noise could not be explained by the duration of transient gamma-band response evoked by the burst noise alone, which was considered as evidence for non-linear relationship between responses by regular and perturbing stimulations. Another magnetoencephalography (MEG) study also reported that responses to regular click-trains with an extra-click halfway, served as the perturbing click, were unaccountable by the linear superimposition (Lutkenhoner and Patterson, 2015). These phenomena were considered as implications that synchronized ASSR was disturbed due to the occurrence of extra stimulus (Ross et al., 2005; Thut et al., 2011; Lutkenhoner and Patterson, 2015).

However, the phenomenon of prediction discrepancy may be ascribed to the unavailability of appropriate templates for transient responses. The implicit transient template constituting the ASSR would differ from the explicit AEP in response to individual stimulus event. This issue was first addressed by Santarelli et al. (1995) who claimed that improved prediction could be achieved using a template of modified last click response (mLCR) to a click train at the same high stimulus rate of the 40 Hz ASSR. The mLCR was found with its NaPa component amplitude smaller (NaPa is the complex wave consisting of the first negative wave Na and the first positive wave Pa in MLR) and its NbPb component amplitude larger (NbPb is the complex wave consisting of the second negative wave Nb and the second positive wave Pb in MLR) than its counterpart from the low stimulation rate at 7.9 Hz. The fact indicated the adaptation effect of the neural system. However, a later study (Presacco et al., 2010) revealed mLCR was not a satisfied template of ASSR either, and suggested a better template from a deconvolution method termed continuous loop averaging deconvolution (CLAD).

The CLAD method is based on the linear convolution model between the transient response to individual stimuli in ASSR and the ASSR stimulus sequence (Delgado and Ozdamar, 2004; Bohorquez et al., 2007; Ozdamar et al., 2007; Wang et al., 2013a). In the classical ASSR paradigm, the isochronic stimulus sequence is used and transient responses to individual stimuli cannot be obtained mathematically from overlapped steady state responses due to the singular nature of inverse problems when the same inter-stimulus intervals (ISIs) are used in the whole sequence. The CLAD method designed an irregular stimulus sequence with variable ISIs, i.e., the ISI-jitter sequencing scheme, which produces quasi-periodic response mimicking the classical ASSR so that the inverse solution to obtain transient response is possible. Such a linear convolution model is based on the superposition mechanism that assumes identical transient responses to stimuli with different ISIs in a sequence.

Utilizing the CLAD method, Ozdamar et al. (2007) studied the effects of stimulus rate (5–98 Hz) on MLRs. Multiple adaptation effects were found on the main MLR components over these rates, e.g., the amplitude decline of wave-Pa with increasing rates, and a wave-Pb resonance at 40 Hz, which might be related to the generation of 40 Hz ASSRs. Later, it was also demonstrated that reconstructed transient responses at 40 Hz could accurately predict the 40 Hz ASSRs in wake or general anesthesia state (Bohorquez and Ozdamar, 2008; McNeer et al., 2009). Moreover, the prediction accuracy could be improved when the ISI-jitter was reduced.

**Figure 1:**
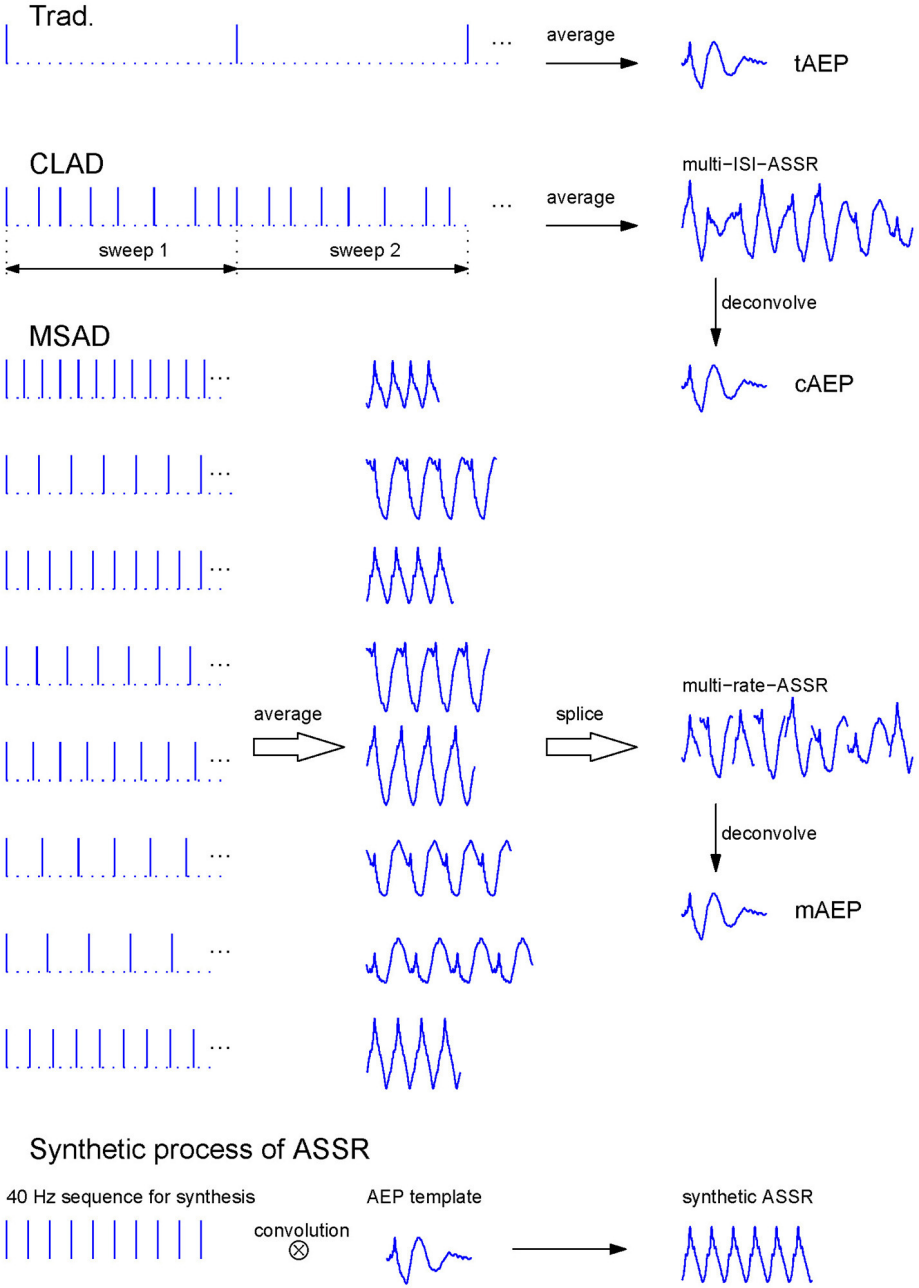
Stimulus sequences, transient AEPs extraction for the Trad., CLAD, and MSAD paradigms, and synthetic process of ASSR by transient AEP template. The Trad. sequence comprises stimuli presented with a large ISI of 204.8 ms (the 1^st^ row). The tAEP was the averaged response to every stimulus. The CLAD irregular sequence (the 2^nd^ row) consists of a number of reduplicative stimulus sweeps with 8 ISI-jittered stimuli each, where ISIs = {16.00, 28.80, 19.20, 27.20, 24.00, 32.00, 36.80, 20.80} ms. Responses elicited by sweeps of stimuli were averaged to gain the multi-ISI-ASSR that was then being deconvolved to obtain the cAEP. The MSAD evenly-spaced stimulus sequence (the 3^rd^ ~10^th^ rows) includes 8 isochronic stimulus sequences with the same ISIs as those in CLAD. These rate-jittered sequences evoked 8 ASSRs. Eight averaged one-cycle ASSRs were spliced one by one into the multi-rate-ASSR similar to multi-ISI-ASSR in CLAD. The mAEP was reconstructed from the multi-rate-ASSR. The bottom showed the synthetic process of ASSR that was the convolution of the transient AEP template with the 40 Hz stimulation sequence with constant ISIs. In this study, three transient AEPs (i.e., tAEP, cAEP, and mAEP) were all used as templates to synthesize corresponding ASSRs, respectively. These synthetic ASSRs will be separately compared with the recorded ASSR in the subsequent analysis.

Recently, Wang et al. (2013b) proposed a new deconvolution approach termed as the multi-rate steady-state average deconvolution (MSAD) method that estimated transient responses from an evenly spaced stimulus-sequencing scheme while based on the same assumption as the CLAD model. The MSAD method does not require jitters in ISI within one stimulus sequence. Instead, it employs the classical ASSR paradigm but with multiple stimulus sequences at different stimulus rates. Basically, the CLAD adopts one stimulus sequence with variable ISIs (ISI-jitter) while the MSAD employs several evenly-spaced stimulus sequences at different stimulus rates (rate-jitter, see Figure 1). In combination with the regularization techniques (Hansen, 1998; Colton et al., 2000), stable solutions for transient response estimation can then be obtained. Therefore, the MSAD used a rate-jitter sequencing scheme, but still assumed identical responses to stimuli at different stimulation rates (Wang et al., 2013b; Tan et al., 2016). Via comparing the CLAD and MSAD paradigms (Tan et al., 2016), it was intriguing to find that deconvolved transient responses at 40 Hz from them showed certain morphological differences. This finding suggested that transient AEPs were not only impacted by the stimulus rate but also by the jittering scheme.

Therefore, while the 40 Hz ASSR can be better predicted using transient responses when the rate effect was taken into account in CLAD, how would the prediction be in the case of using MSAD at the same rate? Would different paradigms manifest differences in the prediction of ASSR? In the present study, the superposition relationship between the classical 40 Hz ASSR and transient responses obtained from 40 Hz CLAD and MSAD paradigms was investigated systematically. Traditional AEPs at a low stimulation rate of 5 Hz were also acquired as a reference template to be compared.

## Materials and methods

### Participants

Twenty-one right-handed normal hearing adults (20–23 years old, 7 females) were recruited in the present study that was carried out in accordance with the recommendations of the human research ethics committee of the Southern Medical University. All participants provided their written informed consents. Two subjects were excluded from the following analysis either because of the lack of a clear ABR or the inconsistency between the odd- and even-trial averages for the purpose of data quality control. No medical history of auditory or nervous system diseases, or psychiatric disorders was reported in all participants. All participants presented audiometric thresholds lower than 25 dB HL obtained with air conduction pure tones at frequencies between 0.25 and 8 kHz. During recording, the subject was instructed to recline and relax in a comfortable chair in a sound-attenuated and electromagnetic-shielded booth.

### Stimulation

Rarefaction clicks of 0.1 ms duration were presented monaurally to the right ear via an insert earphone (ER-3A Ethmotic Research, Elk Grove Village, IL, USA) at 80 dB peak SPL (calibrated in a 2-cc HA2 acoustic coupler using sound level meter). The stimulus sequences and AEP extraction processes for three paradigms are illustrated in Figure 1. The traditional paradigm (Trad. for short in figures and tables) used low stimulation rate at 5 Hz (4.88 Hz exactly) and its AEP was directly extracted via averaging (the 1^st^ row in Figure 1). A sweep of CLAD irregular sequences contained 8 clicks with the ISI = {16.00, 28.80, 19.20, 27.20, 24.00, 32.00, 36.80, 20.80} ms, which was 204.8 ms in length corresponding to a nominal stimulus rate of 39.1 Hz (40 Hz for simplicity) that was defined as the reciprocal of the mean ISIs (the 2^nd^ row in Figure 1). The maximal ISI-jitter of this sequence was 20.8 ms defined as the maximal ISI difference (36.8–16 ms). The stimulus sweep with 8 clicks was delivered to subjects repetitively so that averaging over stimulus sweeps could be performed to attenuate noise. For the MSAD paradigm, several evenly-spaced stimulus sequences with different ISIs were used to produce classical ASSRs at corresponding stimulus rates (Wang et al., 2013b). The ISIs used in the MSAD sequences (3^rd^–10^th^ rows in Figure 1) were same as those in the CLAD paradigm. Therefore, the nominal stimulus rate was the same as in the CLAD. As a control, an isochronic sequence of 25.6 ms ISI was used to achieve classical ASSR at 39.1 Hz (40 Hz for simplicity).

### EEG recording and processing

Electroencephalogram (EEG) was recorded at Fz using Ag/AgCl electrodes with the reference electrode placed on the right mastoid and the ground electrode on Fpz. The electrode impedance was kept below 5 kΩ. EEG data were amplified 10^5^ times (SynAmps^2^ amplifier, Compumedics Ltd, Victoria, Australia), filtered at 10–1,000 Hz, and sampled at 20 kHz.

For each paradigm, more than 1,500 sweeps of EEG were recorded. A sweep of EEG used in the following averaging processing was defined as an epoch of EEG containing response to one click in the traditional or MSAD paradigm. In the CLAD paradigm, a sweep of EEG consisted of responses to a CLAD sequence with eight clicks. A sweep of classical ASSR EEG included four cycles of ASSR. The order of these four paradigms was randomly assigned to each participant.

Raw EEG data and the consequent data analysis (section Comparisons with Statistical Methods) were processed on the MATLAB (2012) platform. A sweep of EEGs with amplitude of any data point more than 40 μV was rejected as artifact. A few sweeps of EEGs at the beginning and the end of every block that may appear during recording for breaks were also excluded from analysis to eliminate the onset and offset effects during stimulations.

The AEP of the traditional paradigm (teamed as tAEP hereafter) was obtained via averaging all sweeps (each of 200 ms starting from the onset of stimulus) of EEGs recorded at 5 Hz of stimulation. All sweeps of EEGs from the CLAD paradigm (each of 204.8 ms starting from the onset of the first stimulus of the sequence) were averaged to yield a quasi-periodical sweep-response (termed as multi-ISI-ASSR in the 2^nd^ row in Figure 1) at a rate of 40 Hz containing irregularly overlapped transient AEPs. By using the CLAD deconvolution method (section CLAD and MSAD Deconvolution Methods), its transient response was reconstructed and termed as cAEP (Figure 1). In the MSAD paradigm, the similar averaging process yielded eight ASSRs corresponding to eight ISIs or stimulus rates (see the middle part of the 3^rd^ ~10^th^ rows in Figure 1). The MSAD deconvolution method (see section CLAD and MSAD Deconvolution Methods) reconstructed its transient response, termed as mAEP (Figure 1), using these eight ASSRs. A separate ASSR of 40 Hz with 4 cycles in one sweep was averaged to yield recorded ASSR (rASSR hereafter).

### CLAD and MSAD deconvolution methods

The basic deconvolution process for the CLAD method can be found in Figure 1. The ensemble averaging was performed over all sweeps left after artifact rejections to produce a multi-ISI-ASSR that is a complex response corresponding to the stimulus sequence defined in individual sweeps as shown in Figure 1. According to the linear superposition hypothesis, the multi-ISI-ASSR with residual noise can be modeled by a circular convolution between the implicit transient AEP and the impulse train within the sweep (Delgado and Ozdamar, 2004). Based on this model the implicit AEP (cAEP in Figure 1) can be readily obtained using an inverse filter derived from the CLAD sequence after being transformed into the frequency domain (Ozdamar and Bohorquez, 2006). However, the solution can be mathematically unstable without appropriate control of the frequency property of the CLAD stimulus sequence. Various measures (e.g., Wang et al., 2006, 2013a) have been proposed to deal with this issue since the first introduction of the CLAD method (Delgado and Ozdamar, 2004). The most important aspect of the CLAD sequence design is the sequence of ISI-jitters and its optimization to avoid unusual amplification of contamination noise in EEG (Ozdamar et al., 2007; Peng et al., 2017). The sequence used in the present study was a well-tuned impulse train containing eight clicks as presented in section Stimulation and Figure 1. The appropriateness of this sequence could be measured by the noise gain factor, C_dec_, defined by Ozdamar and Bohorquez (2006), which was 0.55, or by an improved metric G_dec_ (Peng et al., 2017), which was 0.79. Both values were close to the value from the optimal solution, indicating the applicability of the sequence.

The MSAD method is also based on the convolution model between the implicit transient response and the stimulus sequence that is differently designed as compared with CLAD. The MSAD method introduces a rate jitter among its stimulus sweeps instead of the ISI jitter as in CLAD. As shown in Figure 1, MSAD sweeps contain several evenly-spaced stimulus sequences with different stimulus rates that produce conventional ASSRs at several different rates. Then MSAD offered a deconvolution method to estimate this AEP (mAEP in Figure 1) from the observed multi-rate-ASSRs (Figure 1). The maximal rate-jitter is defined as the maximal rate difference (e.g., 62.5–27.2 Hz in the present study). Reconstruction of transient AEP from the multi-rate-ASSR is a typical inverse computation that usually suffers from the ill-conditioning problem (Hansen, 1998). This problem was addressed by applying the singular-value decomposition and regularization technique (Wang et al., 2013b).

### Comparisons with statistical methods

Firstly, morphologic differences among three transient AEPs (tAEP, cAEP, and mAEP) were analyzed. Their differences were characterized by difference waveforms obtained by subtracting one AEP from another. Statistical significance was tested by a one-sample *t*-test on the amplitude of each data point on the difference waveform. Then, based on the linear superposition hypothesis, three transient AEPs were used as templates to separately convolve the 40 Hz stimulation sequence with constant ISIs to obtain corresponding ASSRs (denoted by tASSR, cASSR, and mASSR accordingly) as shown in the bottom of Figure 1. These synthetic ASSRs were compared with rASSR in both time and frequency domains. In the time domain, a difference analysis similar to the one applied to transient AEPs was performed. Since ASSR is a periodical waveform due to continuous stimulations, ASSRs with a number of cycles can be represented in frequency domain with a few harmonic vectors. ASSRs with four cycles were transformed to the frequency domain via the fast Fourier transform (FFT), where their energies were found mainly in the first three harmonics (40, 80, and 120 Hz for the 40 Hz ASSR). Therefore, a two-element vector (one for real part and another for imaginary part) was adopted to represent one harmonic and a vector with six elements for the first three harmonics (every two-element for one harmonic) was used to represent individual ASSR in the frequency domain. The differences between synthetic and recorded ASSRs were compared using the two-element difference vector (subtracting one vector from another) for each harmonic and the six-element difference vector for entire ASSRs. A one-multivariate-sample Hotelling's *T*^2^ test was performed on these difference vectors across all subjects to test statistical significance, where the multivariate normality of data was tested by a SPSS macro (normtest) developed by L.T. DeCarlo (DeCarlo, 1997).

In addition, contributions of five components (ABR, Na, Pa, Nb, and Pb) in transient AEPs to the ASSR were also analyzed. With reference to the baseline, every component was manually isolated by zeroing other components. This isolated component was subsequently used as a template to synthesize the component-ASSR in order to calculate contribution of every component to the entire synthetic ASSR, which was defined as a root-mean-square (RMS) ratio between component- and synthetic ASSRs.

## Results

### Morphologies of transient and steady-state responses

**Figure 2:**
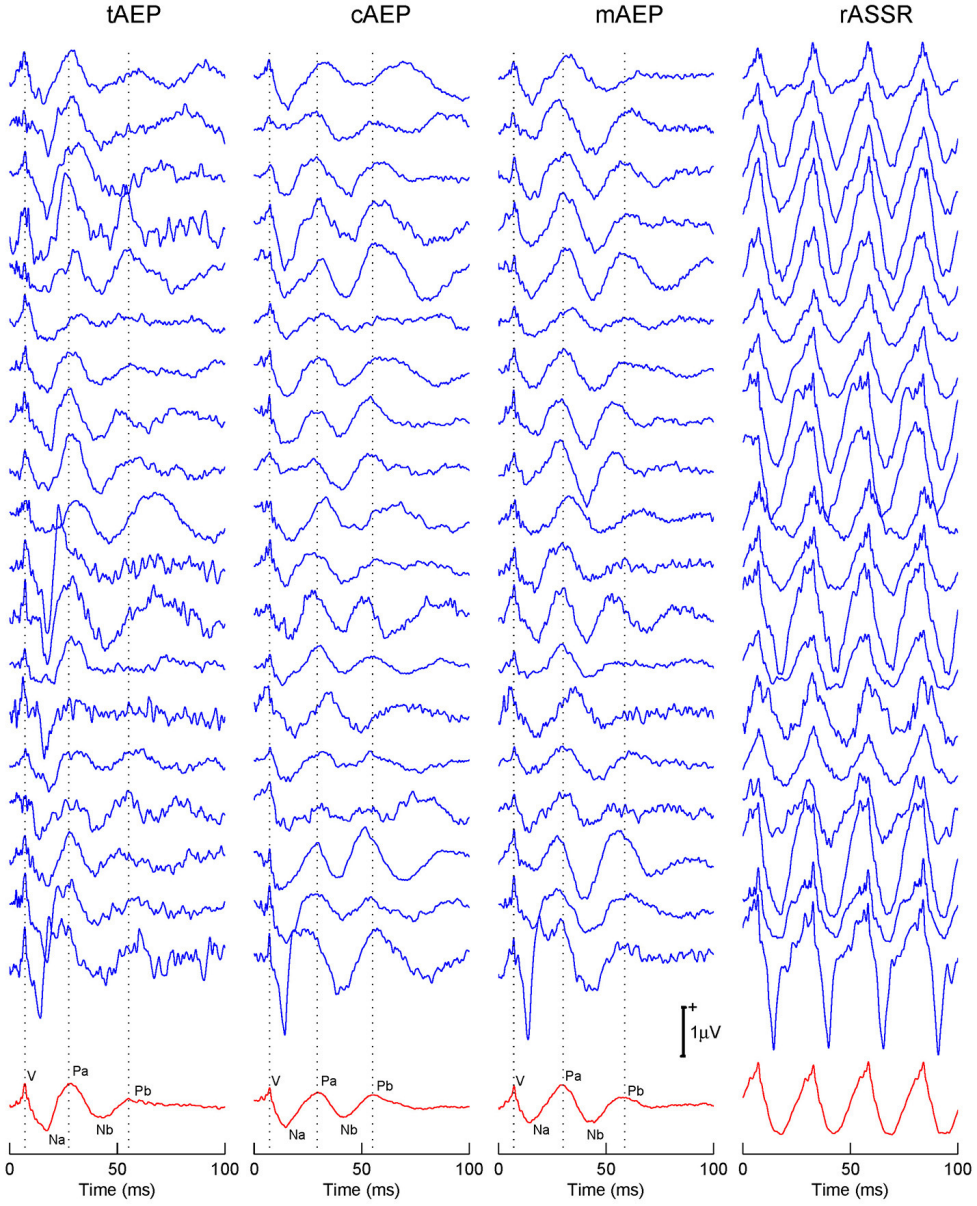
Individual and grand averaged tAEPs, cAEPs, mAEPs, and rASSRs. Sturdy characteristic components (i.e., V in ABR, Na, Pa, Nb, and Pb in MLR) present in the averaged AEPs shown in the bottom row. Three dotted vertical lines label the peaks of V, Pa, and Pb components.

Both recorded and reconstructed transient AEPs and rASSRs of individual participants are shown in Figure 2. Wave-Vs are clearly identified in all transient AEPs, which validate data quality and reconstruction accuracy of transient responses. The tAEPs show more variations and are noisier across individuals than 40 Hz transient AEPs (i.e., cAEPs and mAEPs). Three dotted vertical lines are used to indicate the peaks of three characteristic components (waves-V, Pa, and Pb) with reference to the grand averaged AEPs shown at the bottom. Besides the reliable wave-V, wave-Pa also exhibits relatively stable occurrence and latency in all three transient AEPs, while wave-Pb shows varying appearance and latency, particularly in tAEPs. The four-period rASSRs (the rightest column of Figure 2) show much larger amplitude than all transient AEPs. The grand averages of transient AEPs and rASSRs (the bottom of Figure 2) clearly present all main wave components of ABR and MLR, including waves-V, Na, Pa, Nb, and Pb. Again, the amplitude of the grand rASSR is almost twice as large as the amplitudes of three grand transient AEPs.

**Figure 3:**
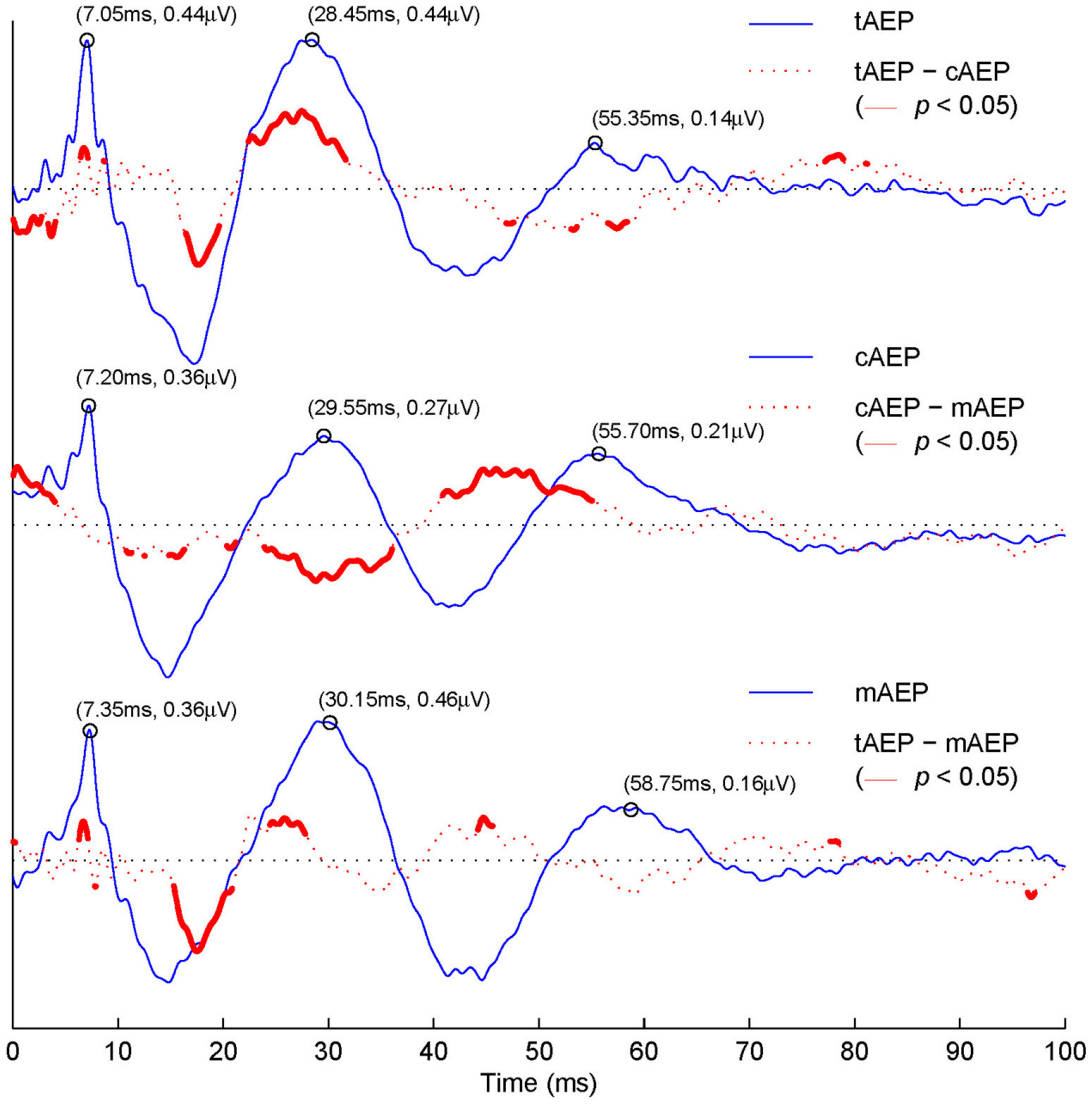
Grand averaged tAEP, cAEP, mAEP, and corresponding paired differences. The 1^st^ row: averaged tAEP (blue solid curve); tAEP–cAEP (red dashed curve), with areas of statistical significant difference (*t*-test, *p* < 0.05) in red bold curves. The 2^nd^ row: cAEP and cAEP–mAEP. The 3^rd^ row: mAEP and tAEP–mAEP.

In Figure 3, difference waveforms obtained via subtracting transient AEPs from two conditions (i.e., tAEP–cAEP in the 1^st^ row, cAEP–mAEP in the 2^nd^ row, and tAEP–mAEP in the 3^rd^ row) are displayed (red dotted curves) together along with transient AEPs (blue solid curves). Furthermore, the areas displayed with thick red curves are those with statistical significant differences between two compared conditions (*t*-test, *p* < 0.05). The values of latency and amplitude for three representative positive waves (V, Pa, and Pb) are labeled in pairs [e.g., (7.05 ms, 0.44μV) for wave-V in tAEP]. The latencies of all three wave-components from both 40 Hz AEPs are prolonged relatively to the latencies of these components in tAEP. Such a phenomenon is in agreement with the previous report that the latencies of AEP components at high stimulation rates (i.e., 40 Hz) are generally longer than those at low stimulation rates (Ozdamar et al., 2007; Valderrama et al., 2014a). Moreover, statistically significant differences are observed in amplitude variations, which can be examined in difference waveforms (red dotted curves). The tAEP and cAEP show significant difference (1^st^ row in Figure 3) at the time windows between ABR and wave-Pb, particularly around the waves-Na and Pa. Significant amplitude differences are also observed between tAEP and mAEP (3^rd^ row in Figure 3) in the range between waves-Na and Pa. The significant discrepancy occurs at the ABR latency range is in fact due to the relatively smaller latency of wave-V in tAEP than mAEP. This phenomenon is also observed in the comparison between tAEP and cAEP (1^st^ row in Figure 3). While differences between AEPs at 5 Hz (i.e., tAEP) and two 40 Hz AEPs (i.e., cAEP and mAEP) are expected, significant differences are surprisingly observed between CLAD and MSAD, mainly in the ranges of waves-Pa, Nb, and Pb (2^nd^ row in Figure 3). It is noted that the difference at the beginning of AEPs comes from a clear drift before the onset of stimuli in cAEP for unknown reasons.

In summary, average transient AEPs demonstrate similar patterns in general but several significant morphological differences among three paradigms. In the following sections, synthesized ASSRs from these transient AEPs are compared against rASSR to characterize the effects of the stimulation rate and sequencing factor on ASSRs in both time and frequency domains.

### Steady-state response synthesis

**Figure 4:**
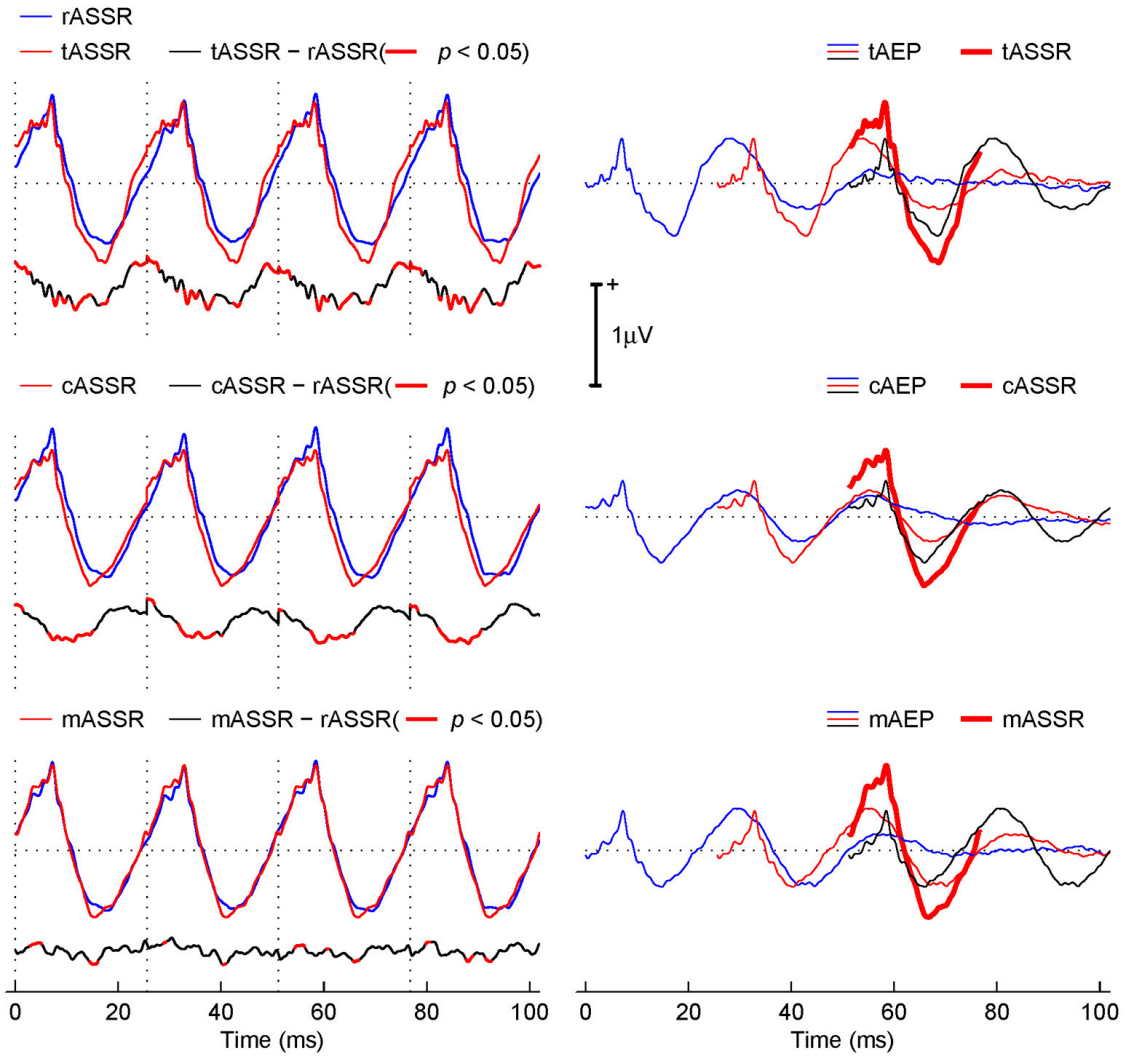
Comparison between the rASSR and tASSR, cASSR, or mASSR. The left column: tASSR, cASSR, and mASSR (red curves) are overlapped with rASSR (blue curves). The difference waveform traces (black curves) are plotted below, where areas of statistically significant difference are depicted in red bold curves (*t*-test, *p* < 0.05). The right column: the linear superposition of three consecutive time-shifted-AEP templates (marked using blue, red, and black curves correspondingly), which is overlapped with the correspondingly synthetic ASSRs (red bold curves).

The left column of Figure 4 shows the synthetic ASSRs (red curves) from three transient AEPs, overlapped with the rASSR (blue curves) for the purpose of comparison. The difference (black curves) between each synthetic ASSR and rASSR is plotted below each ASSR, where red thick areas indicate differences with the statistical significance (*t*-test, *p* < 0.05).

Visually, all three synthetic ASSRs closely resemble the rASSR in general, while the mASSR obviously fits the best. Further inspections lead to observations that tASSR presents a noticeable overestimation in peak-to-peak amplitudes and a phase leading problem (the 1^st^ row in the left column of Figure 4). In contrast, the cASSR also presents the phase leading problem, but the peak-to-peak amplitude was matched except for a minor baseline offset (the 3^rd^ row in the left column of Figure 4). The mASSR is virtually identical to the rASSR except for tiny differences around the negative peaks (the 5^th^ row in the left column of Figure 4). Such observations are supported by difference plots below individual ASSR plots (the 2^nd^, 4^th^, and 6^th^ rows in the left column of Figure 4).

The fact that the difference traces of tASSR and cASSR against rASSR (the 2^nd^ and 4^th^ rows in the left column of Figure 4) exhibits repeated patterns (especially at the portions of significant differences) in line with the periodical ASSRs suggests that real discrepancies occur. Furthermore, these repeated significant differences occupy about 45.95 and 36.28% of the entire response time window of 102.4 ms long (four cycles of ASSR at 39.1 Hz). In contrast, the difference from the MSAD paradigm (the 6^th^ row in the left column of Figure 4) exhibits lower difference magnitude and less repeatable patterns and its significant portions only cover 10.84% of the entire response time window.

The right column of Figure 4 illustrates the synthesis procedure using three consecutive transient AEPs (color coded with blue, red, and black sequentially) from either tAEPs or cAEPs or mAEPs in generating the 3^rd^ period of the corresponding synthetic ASSRs (red thick traces). It is observed that the three major contributors to the formation of the positive part in ASSRs are wave-V (from the black trace), Pa (from the red trace), and Pb (from the blue trace). Although three synthetic ASSRs (red thick traces) do not differ from each other significantly, there seems to be a relatively large difference in the contribution of each component on the composition of ASSRs. As expected, these three main components superimposed in phase at 40 Hz stimulus rate account for the amplitude enhancement in ASSRs. However, amplitude and phase alignments of transient AEP components in the three synthesis procedures are different. For the positive peak in cASSR, waves-V, Pa and Pb are more synchronized than those in mASSR and tASSR where wave-Pa (red traces) leads other two waves (V in black traces and Pb in blue traces). The fact that three components are slightly out of phase in mASSR and tASSR leads to reduced magnitude of the positive peak. However, the relatively large Pa component (in amplitude) in both mASSR and tASSR, compared with cASSR, compensates such a reduction, which leads to the similar positive peak amplitudes in all three ASSRs. It is also observed that the large Na peak amplitude in both tAEPs and cAEPs should account for overestimation of the negative peak amplitude in both tASSR and cASSR, respectively. It is important to note that the ABR component (i.e., wave-V) also contributes to the formation of ASSRs and such a fact suggests that ASSRs contain valuable information from sources in the brain stem. Its delineation from ASSRs might be of significant clinical applications.

### Quantified contributions of transient components to steady-state responses

**Figure 5:**
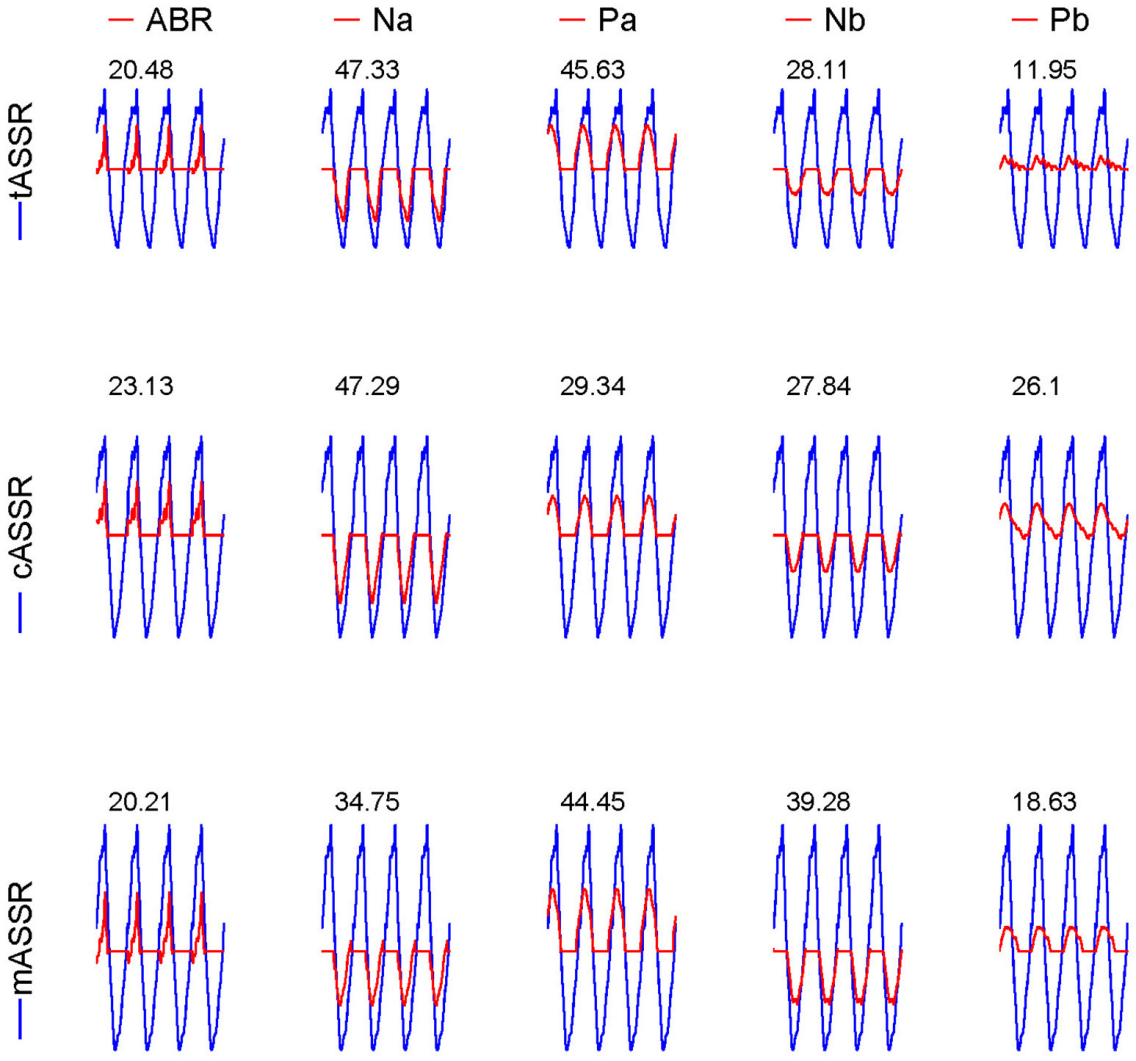
Contributions of AEP components to their corresponding ASSRs. Synthetic ASSRs (blue curves) superimposed by the whole AEPs are overlapped with the synthetic SSRs (red curves) superposed by one of the five components (ABR, Na, Pa, Nb, and Pb) displayed in columns for three paradigms (in rows). The RMS ratios (%) between red SSRs and blue ASSRs are labeled on the top of every subplot.

The contributions of individual AEP components were qualified by separately calculating the RMS percentage of each component (i.e., ABR, Na, Pa, Nb, and Pb) to the entire ASSR (Figure 5). The summation of these ratios over all considered components is usually more than 100% (about 155% in these cases) due to the fact of cancelation among these components during the synthesis procedure. Table 1 shows the percentage values after being normalized toward a total of 100% in order to directly compare different paradigms. Wave-Pa accounts for about 30% in both tASSR and the mASSR, and wave-Na accounts for more than 30% in both tASSR and the cASSR. Noticeably, the contributions of wave-Pb toward both cASSR and mASSR are almost twice as great as that observed in tASSR. Wave-Nb also contributes more in mASSRs (about 25%) than in both tASSR and cASSR (about 18%).

**Table 1:** Contributions of components in three transient AEPs (tAEP, cAEP, and mAEP) to their corresponding ASSRs.

**Components**	**Paradigms**		
	**Trad**.	**CLAD**	**MSAD**
V	13.34	15.05	12.85
Na	30.83	30.76	22.09
Pa	29.73	19.09	28.26
Nb	18.31	18.11	24.97
Pb	7.79	16.98	11.84

*Trad. for traditional low-rate paradigm.The contribution (%) was represented by the RMS ratios between synthesized SSRs with one component and the entire AEPs*.

### Analysis in frequency domain

**Figure 6:**
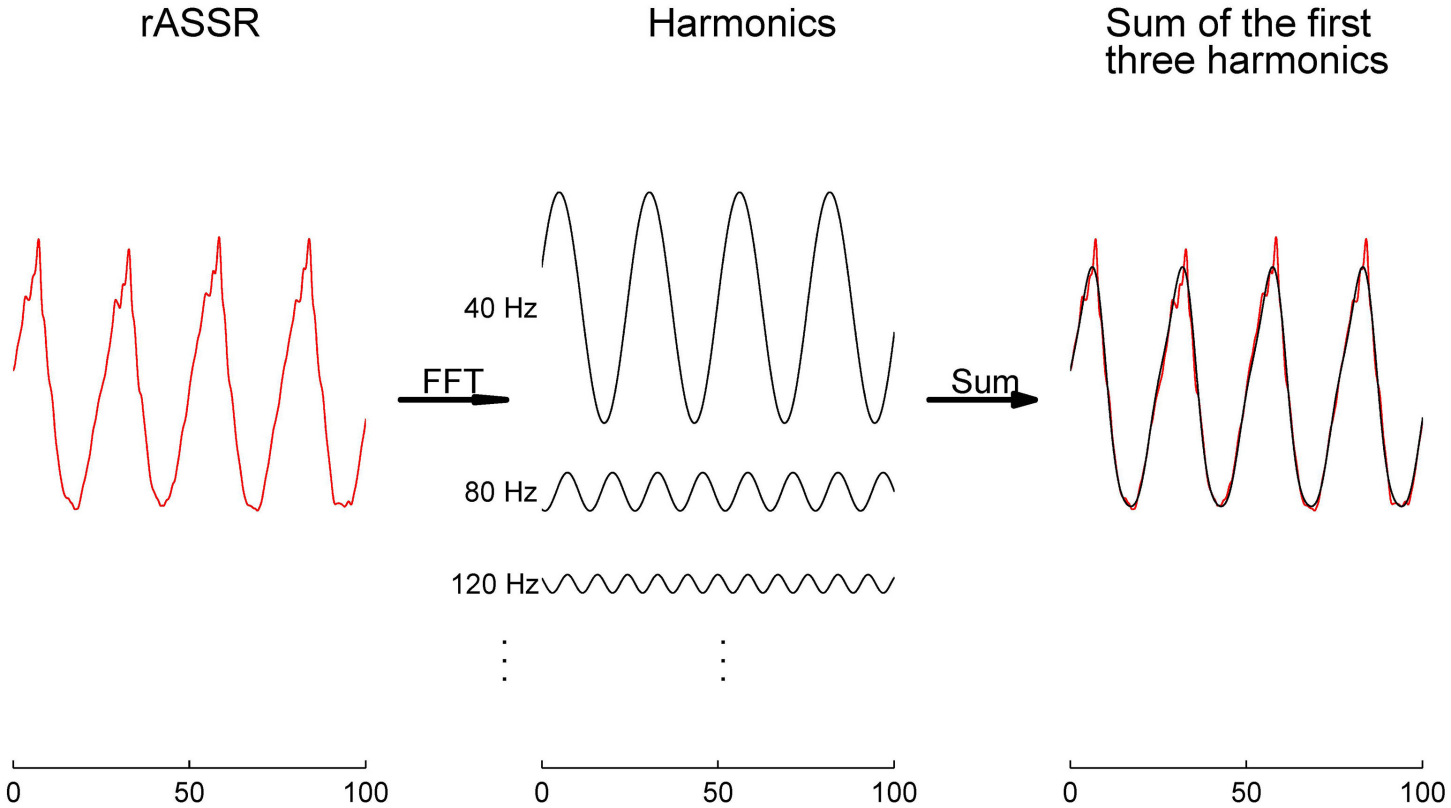
The rASSR approximation by the first three harmonics. The four-cycle rASSR in red was plotted in the left panel. By FFT, the rASSR was decomposed to a number of harmonics (black in the middle panel) at integral multiples of 40 Hz. In the right panel, the summation of the first three harmonics (in black) approximates the original rASSR (in red).

**Figure 7:**
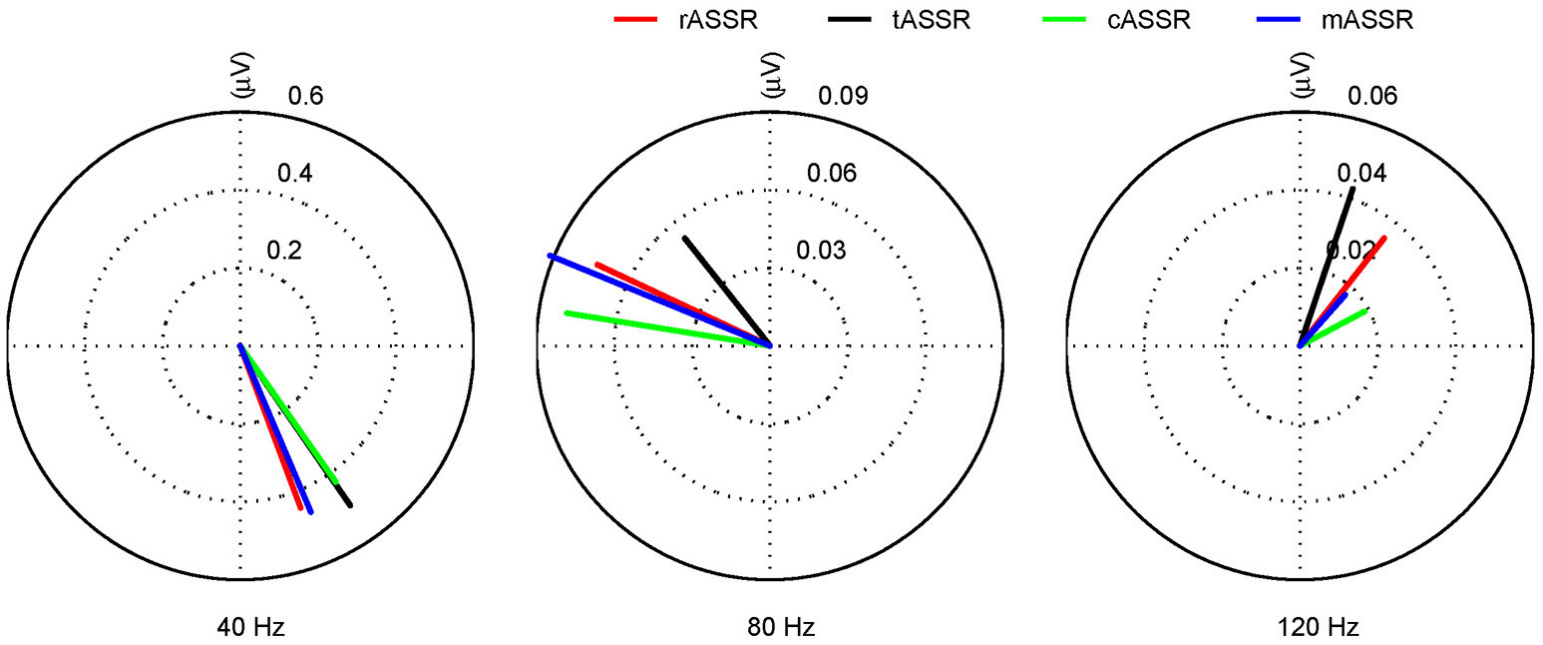
The first three harmonics of tASSR, cASSR, mASSR, and rASSR. All harmonics (40, 80, and 120 Hz) are represented as vectors with RMS amplitude (μV). rASSR: red, tASSR: black, cASSR: green, and mASSR: blue.

rASSR and three synthetic ASSRs were decomposed by FFT to a number of integral harmonics. Taking rASSR as an example, a four-cycle rASSR (the left panel in Figure 6) was decomposed to a group of integral harmonics of 40 Hz as shown in the middle panel. The amplitudes of these harmonics were found to decline with the increasing frequency exponentially. The summation of the first three harmonics (the black curve in the right panel) explained the most variations in rASSR and the reconstruction using the first three harmonics closely resembled the original rASSR (marked with red). Therefore, vectors constituted by amplitudes and angles of the first three harmonics (i.e., 40, 80, and 120 Hz) were used to represent synthetic ASSRs and rASSR to be compared in the frequency domain (Figure 7). These harmonic vectors for different ASSRs are labeled using different colors. The closeness of these vectors in both amplitude and angle indicates the high similarity among these harmonics. Firstly, the 40 Hz harmonics dominate most of signal energies (note the scale difference in Figure 7). The 40 Hz harmonic for mASSR is almost identical to rASSR in both amplitude and angle, while the harmonics for cASSR and tASSR lead slightly to rASSR in terms of phase. The mASSR harmonics at both 80 and 120 Hz also show the closest phase with rASSR in comparison with other ASSRs.

**Figure 8:**
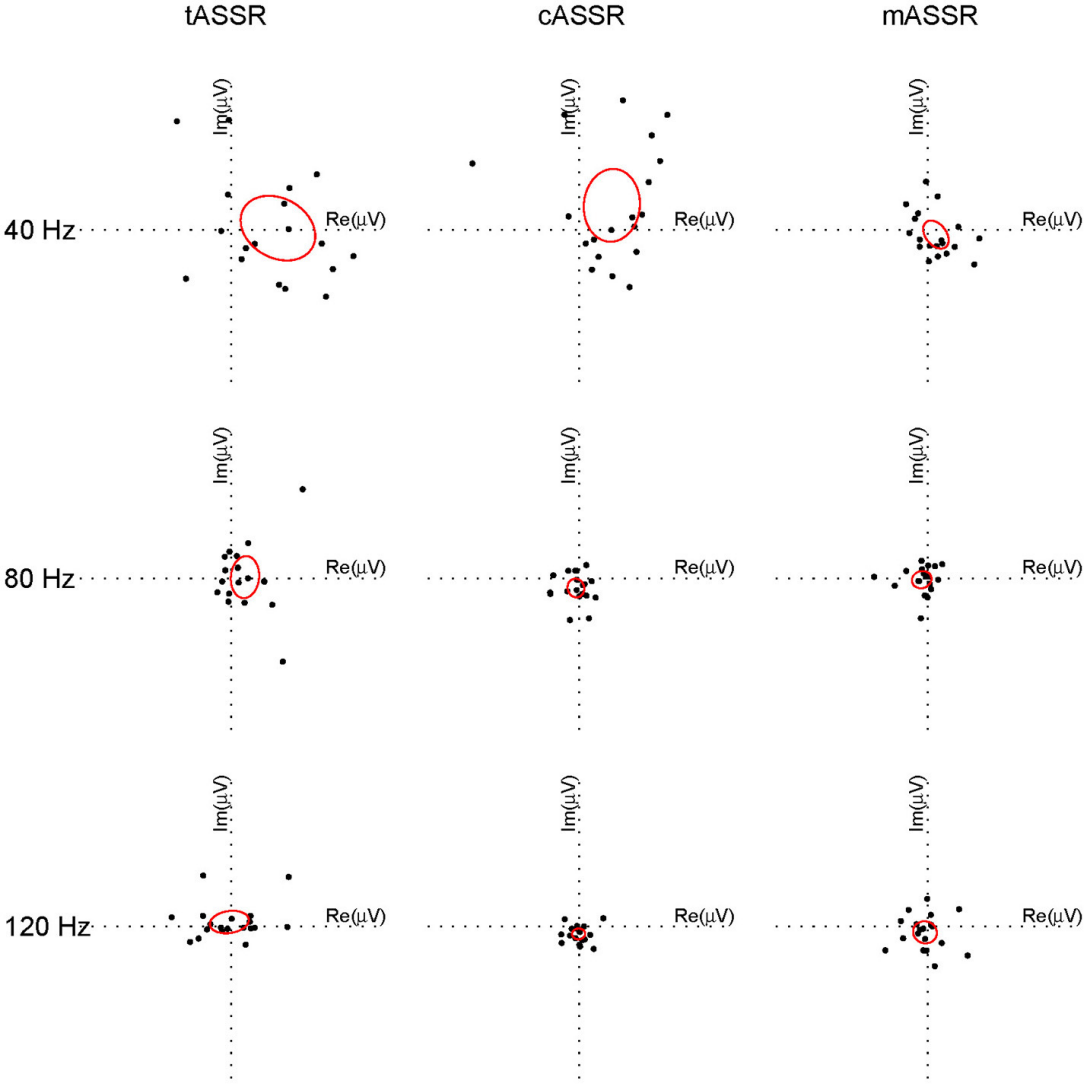
Individual difference vectors between rASSR and tASSR, cASSR, or mASSR at the first three harmonics. Difference vector from each individual out of 19 participants is plotted as a dot in the complex two-dimensional plane (three paradigms in column, three harmonics in row). In a Hotelling's *T*^2^ test, ellipses centered around the tips of averaged difference vectors across all participants are calculated corresponding to *p* = 0.05 for all individual conditions (a harmonic for one ASSR). The origin that is not covered by the ellipse indicates statistical difference between synthetic ASSRs and rASSR at that harmonic.

To evaluate the similarity between synthetic and recorded ASSRs in individuals, difference vectors in the frequency domain represented by a pair of complex values at 40, 80, and 120 Hz were analyzed using the Hotelling's *T*^2^ test. After outliers being removed (i.e., subjects 4 and 11 for the traditional paradigm, subjects 9 and 19 for the CLAD, subjects 12 and 19 for the MSAD), all data were normally distributed (*p* > 0.05). The difference vectors between rASSRs and synthetic ASSRs for each individual (i.e., a dot) are shown in Figure 8, where the confidence limit at each condition (a harmonic for an ASSR) is illustrated using an ellipse that indicates a statistically significant difference (*p* < 0.05, Hotelling's *T*^2^ test), if the origin of the coordinate is not covered by the ellipse (Hotelling, 1931; Picton et al., 1987).

Significant differences to rASSRs were found in cASSRs at three harmonics and tASSRs at 40 Hz harmonics. On the contrast, mASSR matched rASSR at all three harmonics without significant differences. It is demonstrated that mASSR shows the most stable fundamental frequency (40 Hz) measured by the metric of the area of ellipse, indicating the smallest data variance in the complex plane. Quantitative statistical analysis on the overall difference between synthetic and recorded ASSRs (Hotelling's *T*^2^ tests on the vector of 6 elements for difference in complex Fourier coefficients) indicates that significant mismatches to rASSR are only found in cASSR and tASSR (tASSR vs. rASSR: *T*^2^ = 32.33, *F* = 3.70, *p* = 0.029; cASSR vs. rASSR: *T*^2^ = 92.04, *F* = 10.55, *p* < 0.001; mASSR vs. rASSR: *T*^2^ = 6.96, *F* = 0.80, *p* = 0.592).

In summary, the results from the frequency domain analysis suggest that there are significant differences, which mainly come from the 40 Hz harmonic component, in comparing tASSR or cASSR with rASSR, while no significant difference between mASSR and rASSR.

## Discussion

Understanding of the mechanism behind the generation of ASSRs is undoubtedly valuable in facilitating its clinical and basic research applications as biomarkers either for diagnosing brain disorders (Gandal et al., 2010; Rass et al., 2010; Oda et al., 2012; O'Donnell et al., 2013; Isomura et al., 2016; Thune et al., 2016) and/or for understanding fundamental neural processes (Jacoby et al., 2012; Porcu et al., 2014; Lithari et al., 2016). The linear superposition (Galambos et al., 1981; Santarelli et al., 1995; Bohorquez and Ozdamar, 2008; McNeer et al., 2009) and entrainment mechanisms (Ross et al., 2005; Thut et al., 2011; Lutkenhoner and Patterson, 2015) are two main hypotheses for the generation of ASSRs, in particularly at 40 Hz. In the present study, we partially replicated previous recording paradigms (Tan et al., 2016) with newly designed stimulus sequences from different participants to generate the transient AEPs (tAEP, cAEP, and mAEP) which were used as templates to predict the additionally recorded 40 Hz ASSRs. Results showed that only the synthetic ASSR obtained from mAEP succeeded in predicting the 40 Hz ASSR without statistically significant differences. These results quantitatively demonstrated the linear superposition mechanism at 40 Hz when an appropriate transient template was used. The transient template was also found to be affected not only by the stimulus rate but also by the sequencing factor.

Specifically, all three synthetic ASSRs closely resemble rASSR in general, the synthetic tASSR shows the phenomena of peak amplitude overestimation and phase leading problems, the synthetic cASSR shows the phase leading problem only and the synthetic mASSR is almost identical to rASSR in terms of waveform (Figure 4). Such observations are further studied through an exemplar reconstruction of the synthetic procedure of the ASSR waveform and observed differences in tASSR and cASSR are explained by varying magnitudes and phases of transient AEPs from three different paradigms (i.e., classical, CLAD, and MSAD paradigms). The best fit of the mAEP template in generating recorded ASSRs is further supported by results from the quantitative harmonic analysis, together with the Hotelling's *T*^2^ statistical analysis, in which no significant differences of spectral properties are observed in mASSR, but significant spectral differences in tASSR and cASSR, as compared with rASSR. Therefore, optimal ASSR reconstructions can be achieved through the synthesis procedure (the implementation of linear superposition principle) using the MSAD method, while no significant improvements are achieved in reconstructing ASSRs using cAEPs obtained at 40 Hz (mean value) stimulus rate by the CLAD method as compared with the use of tAEPs at 5 Hz by the traditional method.

Significant differences between cAEPs and mAEPs, as well as morphological differences in estimated cASSRs and mASSRs, should be attributed to different stimulus-sequencing schemes used in CLAD and MSAD as there is no real-time ISI jitter in single MSAD sequences. Therefore, these results indicate that transient AEPs are dependent on not only stimulus rate but also sequencing factor (i.e., ISI-jitter & rate-jitter). It is consistent with the previously reported stimulus-sequencing effect on ASSR predictions using the CLAD method with different ISI-jitter sequences (Bohorquez and Ozdamar, 2008), where sequences with lower ISI variations tend to generate more accurate ASSR predictions. The stimulus-sequencing effect has been found in other stimulation modality, e.g., visual protocols. For instance, at the stimulation rate of 7.5 Hz, the discrepancy between recorded visual steady-state response (SSR) and synthetic SSR from the transient template deconvolved from responses evoked by a pseudo-irregular stimulus sequence (m-sequence) has been reported (Heinrich et al., 2015).

It has been found that the NbPb complex appeared to be sensitive to the sequencing scheme. For example, cAEPs obtained from two CLAD sequences as shown in Figure 3 and the results in Tan et al. (2016), the NbPb variation can be observed. Similar variation can be also observed in Ozdamar et al. (2007) and in Holt and Ozdamar (2015), where they failed to find a consistent Pb resonance at 40 Hz. Using a different deconvolution method and stimulus sequences, Valderrama et al. (2014a) reported that the amplitude of the NbPb complex initially declined when the click-stimulus rate increased from 8 to 20 Hz, then enhanced from 20 to 67 Hz, and reduced again from 67 to 125 Hz. In addition, Valderrama et al. (2014b) also reported that ABR may be affected by the ISI distribution in a jittered sequence, which was assumed to be related to the slow mechanism of adaptation (LeMasurier and Gillespie, 2005; Zhang et al., 2007). These results may anticipate further study to address the stimulus-sequencing effects on AEPs. On the other hand, since there are no real-time jitters in MSAD sequences, these effects have been minimized in reconstructing transient AEPs behind classical ASSR protocols.

This sequencing-dependent phenomenon might be caused by the neuronal adaptation mechanism (Picton, 2011; Perez-Gonzalez and Malmierca, 2014). It has been extensively reported that the AEP amplitude declines in repeated auditory stimulations and with increasing stimulation rates are probably indicative of an intrinsic neural mechanism to avoid overstimulation of the auditory system (Ozdamar et al., 2007; Picton, 2011; Valderrama et al., 2014b). This rate-dependent adaptation should be at least one of the reasons for the peak magnitude overestimation in tASSRs, because amplitudes of components in transient AEPs obtained at low stimulation rates (templates used to reconstruct tASSRs) are higher than those from high stimulation rates (rASSRs). On the opposite side, neuronal systems show the phenomenon with quick and vigorous responses to novel stimulations (opposite to repeated stimuli causing adaptation) (Perez-Gonzalez et al., 2005; Malmierca et al., 2009; Costa-Faidella et al., 2011). Variations in acoustic properties of a stimulus element or temporal sequences among stimulus elements can be interpreted as novel information that triggers the suppression of adaptation. In the present study, the ISI is constant for an rASSR stimulation sequence and an MSAD sequence that might lead to adaptation, whereas the CLAD sequences should show less adaptation effects due to the ISI-jitter (as large as 20.80 ms). Therefore, mAEP might be more stable than cAEP, which is consistent with the fact that mAEP has more accurate prediction of ASSR than cAEP. This may also imply that the auditory system is capable of detecting small stimulation changes and cAEP should be intrinsically different from the transient AEP underlying rASSR.

Beyond the superimposition hypothesis, an alternative generation hypothesis of ASSR is the entrainment of a neural rhythm (Ross et al., 2005). Ross et al. (2005) reported that magnetic ASSRs elicited by a train of tones were disrupted by an extra disturbance stimulus of filtered noise, which could not be predicted by superimposing responses to the added separate monaural stimulation. It was suggested that the disturbance stimulus might desynchronize neural activities underlying ASSR, which was endorsed by a recent study of magnetic ASSR (Lutkenhoner and Patterson, 2015). Lutkenhoner and Patterson (2015) found that the response to an isolated click could not be predicted by subtracting responses to an isochronic click train and the same click train with an extra click halfway. A transient disturbance response of about 200 ms was observed to indicate the phase desynchronization. However, these results cannot rebut the superimposition hypothesis since the disturbance stimulation imposes novelty in stimulation sequences and expectedly altered transient neural responses (e.g., disturbance on the adaptation process).

In the present results, both ABR and MLR contribute to ASSR, which suggests the composition nature of ASSR. It is in consistent with the source analysis based on multi-channel scalp recordings, which suggests that the 40 Hz ASSR may mainly arise from the auditory cortex together with a significant brainstem component (Plourde, 2006; Picton, 2011). Ample evidence showed that the brainstem is the generator of ABR while the thalamus and auditory cortex are suggested for MLR (Plourde, 2006; Picton, 2011). Moreover, both ASSR and MLR are reported to dramatically change during anesthesia (Picton, 2011), which also implies their close relationship. Although Plourde and Villemure (1996) found the attenuation of 40 Hz rASSR was much more pronounced than synthetic ASSR by the linear superposition of the ABR/MLR under the influence of enflurane, this may mainly due to the fact that transient ABR/MLR used for synthesis was from low stimulation rate (2.9 Hz) rather than 40 Hz. In another general anesthesia experiment similar to Plourde and Villemure (1996), McNeer et al. (2009) succeeded in predicting the 40 Hz rASSR using the ABR/MLR reconstructed at 40 Hz. Regarding the weights of both components, MLR accounts for the majority of ASSR, which is similar to the one reported in a previous study (Bohorquez and Ozdamar, 2008), although the exact amounts of contributions from these two components are different. Since it is possible for multiple different transient templates in generating a similar synthetic ASSR response due to the ill-posed property of the deconvolution process (Sparacino et al., 2004; Tan et al., 2015), more studies are needed to investigate this issue.

It has been reported that schizophrenia patients can be characterized by auditory perceptual abnormalities. A meta-analysis of data from 1999 to 2016 shows that schizophrenia patients exhibit a robust reduction of spectral power and phase synchronization in the 40 Hz ASSR (Thune et al., 2016). Such an abnormality of the 40 Hz ASSR has been suggested in relation with the dysfunction in parvalbumin γ-aminobutyric acid (GABA) interneurons (Sohal et al., 2009; Lewis et al., 2012) in which inhibiting parvalbumin interneurons was reported to produce a reduction of rhythmic activity in the 40–70 Hz range, and/or N-methyl-D-aspartate (NMDA) receptors (Kantrowitz and Javitt, 2010; Nakao and Nakazawa, 2014; Sivarao et al., 2016), where NMDA channel occupancy was found to lead to inverse modulation of 40 Hz ASSR. Thus, ASSR can be a valuable translational biomarker for schizophrenia and related disorders, although the characterization of ASSR in relation to the development and course of disease has not been adequate (Thune et al., 2016). The same study also claimed that electrophysiological indices from AEPs, such as mismatch negativity or Pb, are more sensitive to dysfunctions in schizophrenia than ASSR (Thune et al., 2016). Now, with the capacity of obtaining ASSR and its underlying transient components, such as Pb, simultaneously (empowered by the MSAD method), it can potentially help avoid the competitive comparison between ASSR and AEP components on diagnosing brain disorders. Furthermore, concurrent information from both ASSR and AEP might reveal sources of abnormalities in neuropsychiatric disorders (e.g., schizophrenia) more precisely than those from each individual. Source identification of abnormalities may even help to identify targets for treatment in patients, while all of these need further studies.

In the present study, the relationship between ASSRs and their transient responses was investigated only under the steady-state condition, where the onset and offset responses to stimulus sequences were carefully removed. This is due to the limitations of the adopted deconvolution techniques. The CLAD and MSAD methods are based on the assumption that individual responses to every stimulus in the stimulus sequence are same. This requirement can be approximately achieved under the steady-state condition, while variations at the onsets and offsets of stimulus sequences might violate the assumption. Other experimental techniques are needed to address the linear superposition hypothesis under the non-steady-state condition, such as at the onset and offset.

In clinic, ASSR is usually elicited by modulated stimuli, which are continuous and able to simulate real sounds from environments. Click stimuli, utilized in the present study, is however one kind of pulse stimulations that is different from real-life sounds. Therefore, the future work may focus on how to reconstruct transient AEP from ASSR evoked by modulated stimuli (e.g., stimuli with modulated amplitude and/or frequency).

## Conclusions

In the present study, the use of transient responses from the 40 Hz mAEP succeeds in predicting the 40 Hz rASSR, which strongly supports the linear superposition generation mechanism of ASSR. The finding is important in the advancement of the potential use of ASSR in clinical and basic research. The MSAD method is the technique behind that is able to reconstruct the precise transient response underlying the 40 Hz ASSR since MSAD uses a similar stimulus-sequencing scheme as in the classical ASSR and addresses the ill-conditioned inverse problem at the same time. As a result, the unique merging of obtaining the ASSR and its underlying transient responses at the same time provides valuable insights about activations and sequence of activations in the auditory pathway structures.

## Ethics statement

This study was carried out in accordance with the recommendations of the human research ethics committee of Southern Medical University with written informed consent from all subjects. All subjects gave written informed consent in accordance with the Declaration of Helsinki. The protocol was approved by the human research ethics committee of Southern Medical University.

## Author contributions

XT, QF, and TW conceived and designed the study, performed the experiments, analyzed and interpreted the data, and wrote the manuscript. HY and LD conceived and designed the study, interpreted the data, and wrote the manuscript.

### Conflict of interest statement

The authors declare that the research was conducted in the absence of any commercial or financial relationships that could be construed as a potential conflict of interest.

## Abbreviations

cAEP: transient auditory evoked potential reconstructed by continuous loop averaging deconvolution method
cASSR: synthetic auditory steady-state response superimposed by the transient auditory evoked potential reconstructed by continuous loop averaging deconvolution method
CLAD: continuous loop averaging deconvolution
ISI: inter-stimulus interval
mAEP: transient auditory evoked potential reconstructed by multi-rate steady-state average deconvolution method
mASSR: synthetic auditory steady-state response superimposed by the transient auditory evoked potential reconstructed by multi-rate steady-state average deconvolution method
mLCR: modified last click response
MSAD: multi-rate steady-state average deconvolution
rASSR: classically recorded auditory steady-state response
SSR: steady-state response
tAEP: traditional transient auditory evoked potential at 5 Hz
tASSR: synthetic auditory steady-state response superimposed by traditional transient auditory evoked potential at 5 Hz.
